# X-ray powder diffraction in education. Part II. Intensity of a powder pattern

**DOI:** 10.1107/S1600576723002121

**Published:** 2023-05-09

**Authors:** Robert Dinnebier, Paolo Scardi

**Affiliations:** a Max Planck Institute for Solid State Research, Heisenbergstrasse 1, Stuttgart, 70569, Germany; b Department of Civil, Environmental and Mechanical Engineering, University of Trento, via Mesiano 77, Trento, 38123, Italy; Wilfrid Laurier University, Waterloo, Ontario, Canada

**Keywords:** powder diffraction, peak intensity, intensity corrections, powder patterns, *Mathematica*

## Abstract

This article is the second part of a series dealing with the description and visualization of mathematical functions used to describe a powder diffraction pattern for teaching and education purposes. The first part dealt with the instrumental and sample contributions to the profile of a Bragg peak [Dinnebier & Scardi (2021 ▸). *J. Appl. Cryst.* **54**, 1811–1831]. The second part, here, deals with the mathematics and physics of the intensity in X-ray powder diffraction. Scholarly scripts are again provided using the Wolfram language in *Mathematica*.

## Abstract 2

The most commonly used functions for calculating or correcting step-scan and integrated intensities of a powder diffraction pattern are presented in an educational manner with the support of *Mathematica* scripts. The scripts can be easily used by interested readers to explore the effects of different instrumental and sample parameters.

## 1. Introduction

This series of papers deals with the description and visualization of mathematical functions used to describe a powder pattern. Accompanying each part is a collection of user-friendly, interactive and freely distributable *Mathematica* (Wolfram Research, https://mathworld.wolfram.com/) teaching scripts. All scripts have been written in Wolfram *Mathematica*, version 13.0.0.0, and are constantly updated. They are freely available at the website https://www.fkf.mpg.de/xray. Non-subscribers of *Mathematica* can run the scripts using the freely available *Wolfram Player* at https://www.wolfram.com/player/. Bugs and problems should be reported to r.dinnebier@fkf.mpg.de. In particular, the ‘Manipulate’ command is extensively used to visualize the impact of parameters in an interactive manner. When possible, parameter values from real-life examples are given as the default inputs. The idea is to ‘learn by doing’; one may gain intuition for how a given mathematical model performs for describing diffraction peaks in an experimental powder pattern and what the limitations of the said model are. Every model is an oversimplification of the underlying physics, but different models can be useful for studying various phenomena or increasing the precision of the investigation.

We begin by introducing the scripts that visualize the complex atomic form factor for angular- and energy-dispersive X-ray diffraction and the displacement factor due to thermal motion[Sec sec3]. This is followed by a discussion of the complex structure factor and the effect of thermal diffuse scattering on a powder pattern. Then, a series of correction factors for step-scan and integrated intensities are discussed in detail, including the Lorentz and polarization factors, multiplicity, various absorption effects, the overspill effect, and preferred orientation. Finally, the intensity distribution of a powder pattern is demonstrated for a nanocrystalline material, following two alternative approaches based on (i) the structure factor and common volume function (CVF), including the effect of small-angle scattering for spherical particles, and (ii) total scattering from a single crystallite, with atomic distances used in the Debye scattering equation.

In general, the scattering vector is given as d*, **q** or **s**, which are used interchangeably given the following relations:

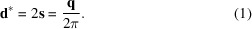

The corresponding lengths are defined as

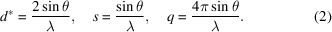

The scattering angle is 2θ and λ is the wavelength of the X-ray beam. At a Bragg reflection, the Bragg condition is given as

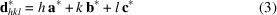

with the integer indices *hkl* (called Miller indices if coprime) and the reciprocal-lattice parameters a*, b* and c*.

## 2. The atomic form factor

The atomic form (or scattering) factor *f*_*j*_ describes the scattering power of an atom or ion *j* as a function of the length of the scattering vector assuming spherical symmetry of the electron cloud. In the case of X-rays, the form factor depends strongly on *s* with a marked decrease at higher values. The value at *s* = 0 is normalized to the number of electrons of the scatterer (atom or ion). The difference between the ionic and atomic form factors is small and barely visible in powder diffraction patterns, except for compounds showing mainly ionic character and containing lighter elements like Na^+^, F^−^, O^2−^ or Al^3+^. The form factor consists of a term that depends on the distance in reciprocal space (normal scattering) and a complex part depending only on the wavelength (anomalous scattering):

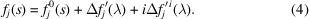

**Figure 1:**
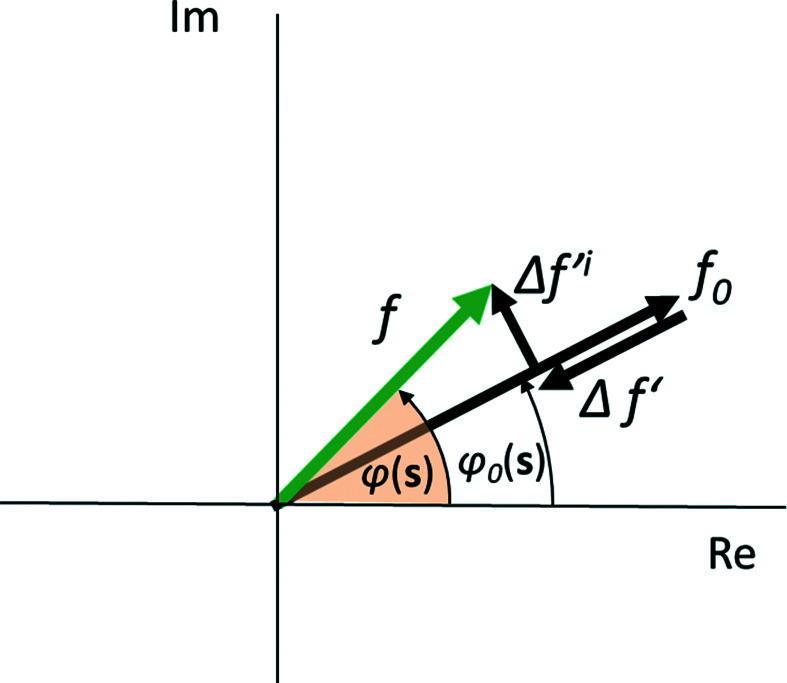
Vector (pointer) representation of the complex atomic scattering factor *f* with normal scattering (*f*_0_) and real (Δ*f*′) and imaginary (Δ*f*′^*i*^) parts of anomalous scattering [from Dinnebier *et al.* (2018 ▸)]. Δ*f*′ is assumed to be negative. The phase angle φ changes in the presence of a non-negligible imaginary (Δ*f*′^*i*^) component of the anomalous scattering.

The real part [the term 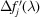 ] of the anomalous scattering factor has a phase shift of π with respect to the normal scattering factor, thus keeping the original phase angle of 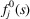 . Since the value is predominantly negative, it reduces the scattering power in most cases. [For light elements and away from absorption edges 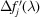 can be (small) positive.] In contrast, the imaginary part [the term 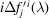 ] has a phase shift of π/2 (Fig. 1 ▸), leading to both a change in the absolute value of the form factor

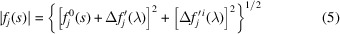

and a shift of the original phase of 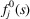 by

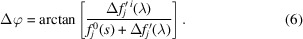

**Figure 2:**
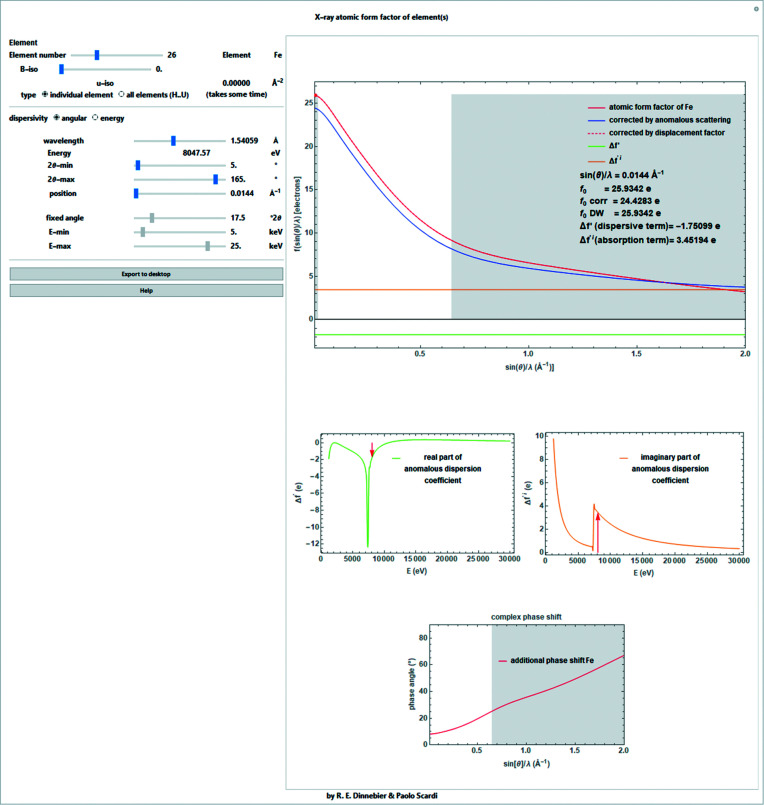
Screenshot of a *Mathematica* script for determining the individual atomic form factor for fixed-wavelength data for atoms ranging from order number 1 (H) to 92 (U) [*e.g.* iron (*Z* = 26) is shown here]. Upper chart: absolute form factor without correction for anomalous scattering (red line), corrected for anomalous scattering (blue line), corrected for Debye–Waller factor (introduced in Section 3) (red dashed line), the real part of the anomalous dispersion coefficient (green line) and the imaginary part of the anomalous dispersion coefficient (orange line) depending on the scattering length *s*. Lower charts: real and imaginary parts of the anomalous dispersion coefficient and the complex phase shift introduced by the imaginary part of the anomalous dispersion coefficient. The non-accessible range for selected wavelength and angular ranges is shaded in gray.

The functional dependence of the form factors for all common atoms and ions has been parameterized by an empirical linear combination of five Gaussian functions:

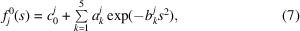

with the 11 parameters *a*_1_…*a*_5_, *b*_1_…*b*_5_ and *c*_0_ tabulated *e.g.* by Waasmaier & Kirfel (1995 ▸). The resulting form factors are valid over the scattering range 0 ≤ *s* ≤ 6 Å^−1^ (Fig. 2 ▸).

Anomalous scattering effects are often disregarded for simplicity, but they become extremely important if the wavelength used is in the vicinity of an absorption edge of an atomic species in the sample. For a strong scatterer, the change in scattering power can amount to the equivalent of several electrons and anomalous dispersion measurements can be used to give extra element-specific information about the structure (Fig. 2 ▸).

**Figure 3:**
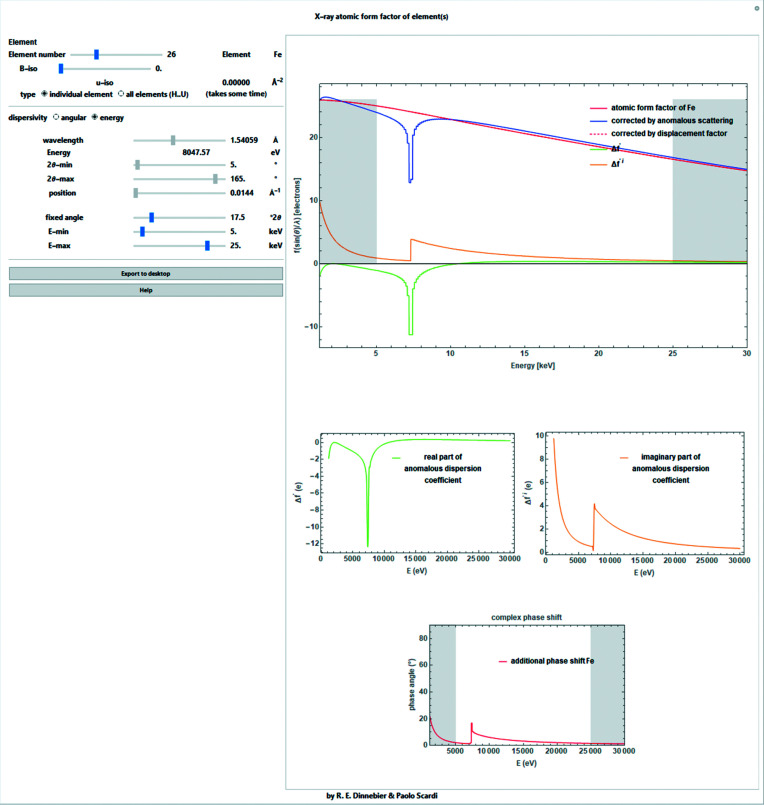
Like Fig. 2 ▸, but as a function of energy.

When using an energy-dispersive measurement geometry with a fixed 2θ angle, equation (4) becomes especially important if the powder pattern runs over absorption edges (Fig. 3 ▸), which is a likely occurrence. The conversion between the length of the scattering vector and the energy in eV is given as

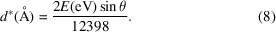

**Figure 4:**
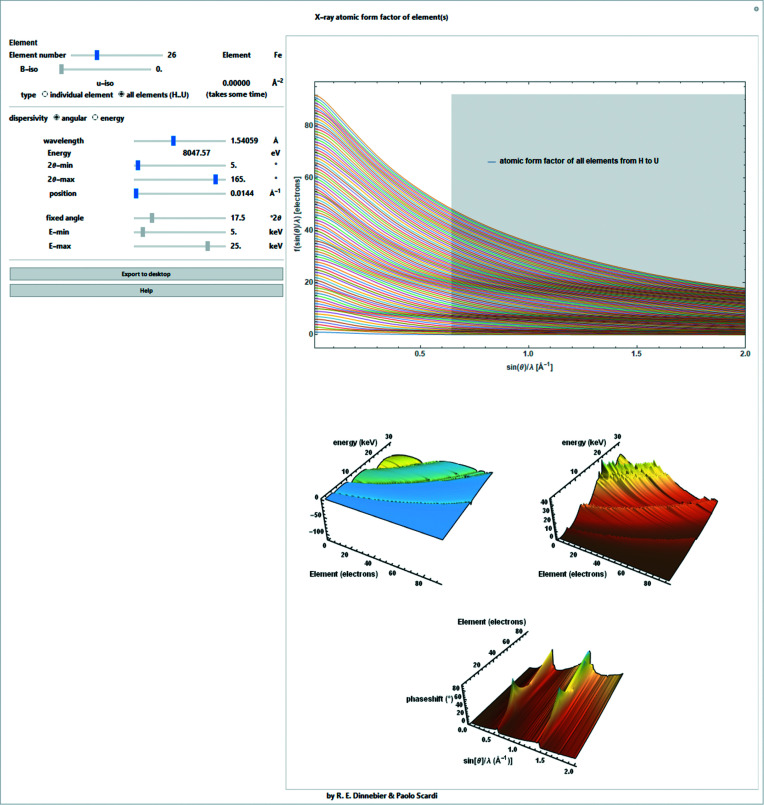
Screenshot of a *Mathematica* script for determining the individual atomic form factor for fixed-wavelength data for atoms ranging from order number 1 (H) to 92 (U) displayed simultaneously. Upper chart: absolute form factor without correction for anomalous scattering depending on the scattering length *s*. Lower charts: real and imaginary parts of the anomalous dispersion coefficient and the complex phase shift introduced by the imaginary part of the anomalous dispersion coefficient.

The *Mathematica* script also allows the simultaneous representation of the scattering factors, anomalous dispersion factors and phase shifts for all elements ranging from H to U (Fig. 4 ▸).

## 3. Atomic displacement parameter

At any temperature, atoms vibrate about their equilibrium position. Moreover, static local atomic displacements may exist in disordered structures like solid solutions or deformed crystals. The corresponding displacements lead to a decrease in peak intensities and an increase in the background due to thermal diffuse scattering. The decrease in peak intensity is described by multiplying the atomic form factor with either an isotropic or an anisotropic correction factor, depending on the scattering length or scattering vector. For the isotropic case, the displacement factor (Debye–Waller factor) for the entire crystal structure, groups of atoms or an individual atom is defined with a dependence on the scattering length *s* as

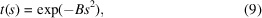

where *B* is the isotropic displacement parameter (typically in Å^2^). The displacement factor is multiplied by the form factor, thus further reducing the scattering power. With single-crystal data, or sometimes with extremely high quality powder diffraction data measured over an extended *s* range, it may be possible to refine anisotropic displacement parameters for the strong scatterers in the crystal structure. The anisotropic displacement parameter can be geometrically described as a three-axis ellipsoid and can be described in terms of a symmetric second-rank tensor:

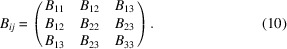

The isotropic analog can be calculated as 1/3 of the tensor’s trace ignoring the off-diagonal parts:

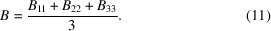

The anisotropic displacement factor depends on the direction of the scattering vector:

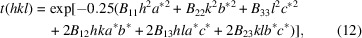

or, in a dimensionless fashion,

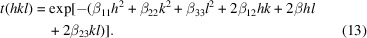

The physical meaning of *B* is given by

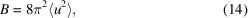

where 〈*u* ^2^〉 is the mean-square deviation from the equilibrium position of the atom or group of atoms, projected along the scattering vector **s**. In the anisotropic case, this leads to

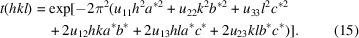

In order to prevent physically meaningless results, the *u*_*ij*_ (or *B*_*ij*_ or β_*ij*_) matrix must be kept positive definite, which can be achieved with the following boundary conditions:

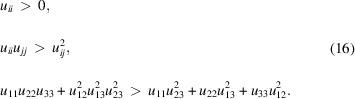

All descriptions of the thermal effects, whether one uses *B*, β or *u*, in isotropic or anisotropic forms, are time averages since the sampling frequency in diffraction measurements is much lower than the characteristic vibrational frequencies.

**Figure 5:**
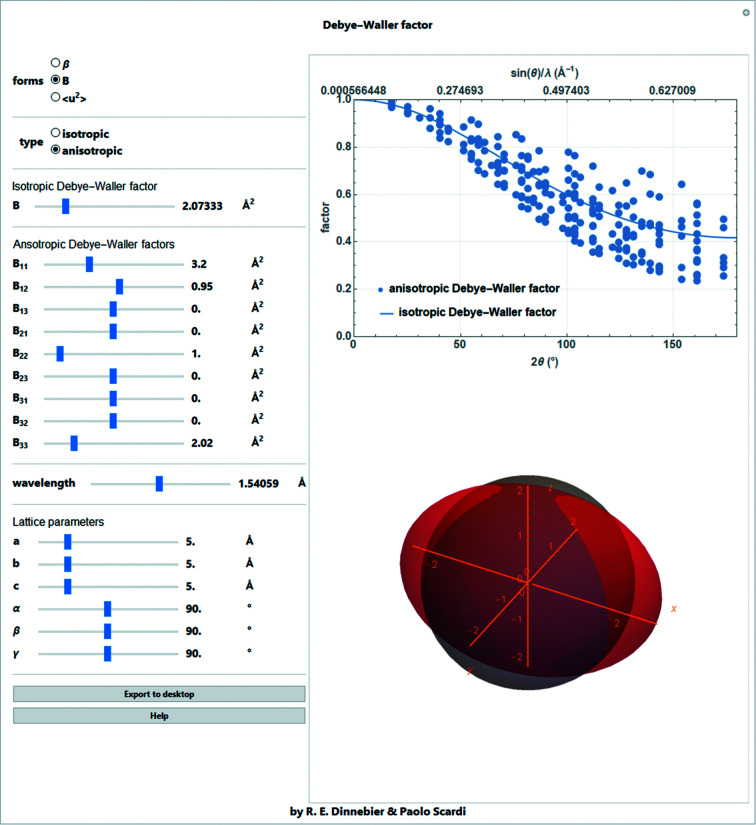
Screenshot of a *Mathematica* script for determining the intensity reduction as a function of *s* and scattering angle for isotropic or anisotropic displacement parameters (*B*, β, 〈*u* ^2^〉). The three-axis ellipsoid representing the anisotropic and the corresponding spherical isotropic displacement parameters is also shown.

In order to determine individual displacement parameters with good precision from powder data, a large range of *s* must be covered. Great care must be taken in interpreting the resulting values, because the *s* dependence of the intensity reduction (Fig. 5 ▸) is similar to that of many other correction factors, which are often poorly treated. (It should be noted that the use of neutrons may give more reliable results.)

Debye’s equation (9) uses the most common and simple approach with the underlying assumption of harmonicity and independent vibrations. Higher levels of complexity using an anharmonic approximation of the atomic displacement parameters are not of relevance for powder diffraction, except in very special cases (*e.g.* Wahlberg *et al.*, 2016 ▸).

## 4. The structure factor

**Figure 6:**
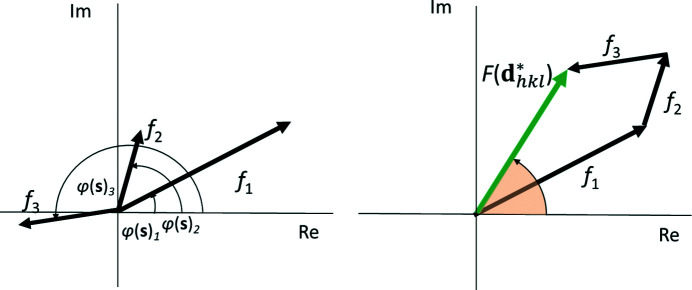
Graphical representation of the complex structure factor as a vector sum (right) of the individual form factors (left) [from Dinnebier *et al.* (2018 ▸)].

The structure factor of a Bragg reflection is defined as a complex sum over all atoms *j* in the unit cell (Fig. 6 ▸):

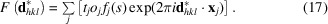

[Commonly, the structure factor is calculated for the Bragg position of the peaks, thus 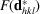 and not *F*(d*). This choice is discussed further later – see equation (57) and related text.] The positional vector *x_j_* of an atom *j* in the unit cell is defined by the fractional crystal coordinates:

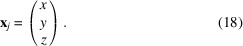

Equation (17) includes for every atom *j* its displacement factor *t_j_* [equation (9)] and a factor *o*_*j*_ denoting the relative occupancy of the atomic site. Both factors are set to unity in the following for simplicity.

**Figure 7:**
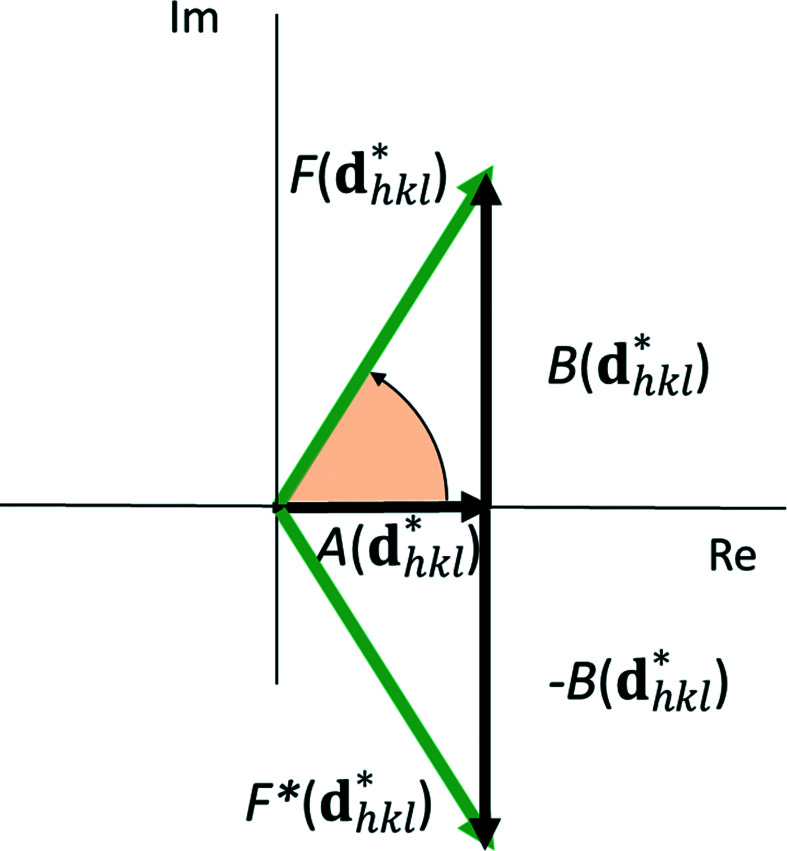
Vector (pointer) representation of the structure factor and its complex conjugate [from Dinnebier *et al.* (2018 ▸)].

Using the Euler identity, the real and complex parts of the structure factor can be separated (Fig. 7 ▸):

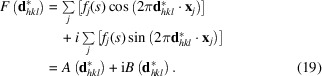

If anomalous scattering is taken into account, the structure-factor amplitude becomes

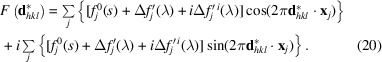

After separating the real and imaginary parts, this further turns into

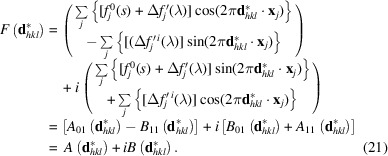




## 5. The intensity of a Bragg reflection

The (integrated) intensity of a Bragg reflection is proportional to the structure factor multiplied by its complex conjugate, which is equivalent to the squared absolute value of the structure-factor amplitude 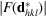 :

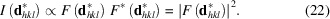

For practical purposes, it is easier to separate the real and imaginary parts of the structure factor (Fig. 7 ▸), leading to

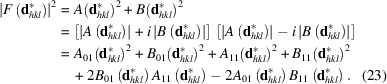

The phase angle of the structure factor can be directly deduced from Fig. 7 ▸ as

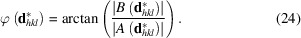




## 6. Thermal diffuse scattering

As the Debye–Waller (DW) coefficient increases, intensity from the Bragg peaks is transferred to the thermal diffuse scattering (TDS). Here a simple TDS model assuming Einstein (independent) oscillators is used (Warren, 1953 ▸; Beyerlein *et al.*, 2012 ▸). For the case of spherical monoatomic nanocrystals of *N*_at_ atoms, the contribution to the background depending on *q* can be simulated by

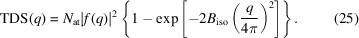

**Figure 8:**
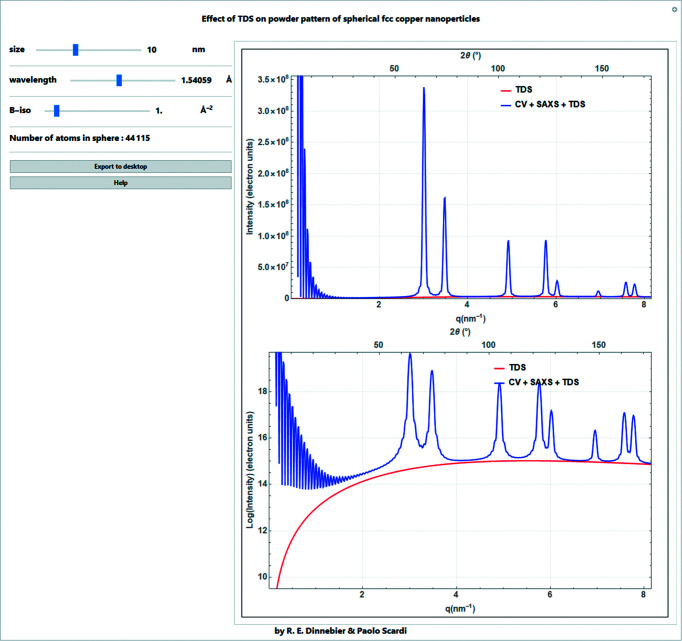
Screenshot of a *Mathematica* script for determining the effect of the isotropic DW factor on peak intensity and of TDS on the background of the powder pattern of spherical f.c.c. copper nanoparticles. The TDS [equation (25)] is the ‘Debye TDS’, which is a TDS model for Einstein oscillators. The powder pattern is calculated with the common volume function [equation (57)] for a fully (linearly) polarized beam and corrected by the Lorentz factor [equation (30)] and an isotropic Debye–Waller factor [equation (9)]. A term to account for small-angle X-ray scattering (SAXS) is applied. A description of these terms is found later in the text.

The effect of the isotropic DW factor on peak intensity and of TDS on the background exemplified in the powder pattern of spherical face-centered cubic (f.c.c.) nanocrystalline copper is illustrated in the *Mathematica* script represented in Fig. 8 ▸. The simple TDS model here has been chosen to illustrate that ‘what is lost’ in the Bragg intensity due to the Debye–Waller factor is transferred to the TDS. This shows that TDS is not ‘background’, but physical information, and that intensity is not ‘lost’.

## 7. Intensity correction factors

The intensities visible in a powder diffraction pattern are altered by a series of correction factors, depending on diffraction geometry, sample preparation and the investigated material. Some correction factors depend on 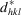 (integrated Bragg intensities) and are therefore constant over the profile of a Bragg reflection. Other corrections can have a d* dependency (step-scan intensities). Taking account of the d* dependency is usually only needed for samples with very broad peaks extending over a wide d* range. Examples of the first type include the multiplicity of a reflection given by the lattice symmetry or the existence of preferred orientation of crystallites in the sample scattering volume. Examples of the second type include the absorption correction, the (solely geometrical) Lorentz–polarization factor and the overspill effect. In the following, the most relevant correction factors are discussed in detail.

### 7.1. Multiplicity

**Figure 9:**
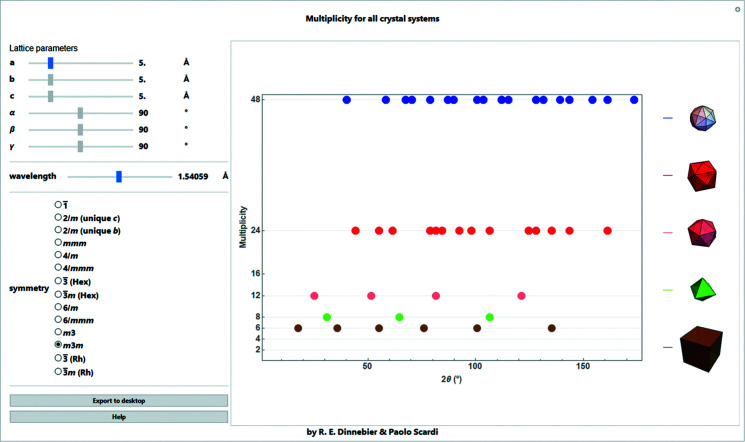
Screenshot of a *Mathematica* script for determining multiplicities – they are given depending on Laue group and lattice parameters as a function of 2θ for a given wavelength. The corresponding closed geometric forms of the Bragg reflections are given for a cubic crystal system.

Because of projection of the three-dimensional reciprocal space onto a direction in reciprocal space, a one-dimensional d* axis in powder diffraction, all planes with identical interplanar spacing will result in an exact superposition of their intensity contributions. This results in a single observed peak. Among these superpositions are ‘systematic overlaps’, which denote symmetry-equivalent reflections with identical intensities. The corresponding factor, called the multiplicity, is an integer which depends on the type of reflection and the Laue group (Giacovazzo *et al.*, 2011 ▸). Because of the overlap of Friedel pairs (Giacovazzo *et al.*, 2011 ▸), the observed intensity is always doubled, corresponding to a minimum value of 2 for the reflection multiplicity for all crystal systems. In addition, for symmetries higher than triclinic, the multiplicity lies between 2 and 48. As an example, the *h*00 reflection for cubic Laue symmetry is a face of a cube. Since all six faces of the cube are symmetry equivalent (*h*00, 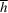 00, 0*h*0, 0 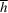 0, 00*h*, 00 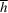 ), the multiplicity is 6. In general, the symmetry-equivalent planes can be visualized as faces in either open or closed polyhedra. A table of all multiplicities for all Laue groups is given *e.g.* by Klug & Alexander (1974 ▸) and Rouse & Cooper (1977 ▸). Multiplicities depending on Laue group and lattice parameters as a function of 2θ for a given wavelength are shown in Fig. 9 ▸. The corresponding closed geometric forms of the Bragg reflections are shown for the case of a cubic crystal system.

### 7.2. Lorentz–polarization factor

#### 7.2.1. Lorentz factor

The Lorentz factor is a purely geometric factor that depends on the scattering angle. It has three contributions (*L*_1_, *L*_2_, *L*_3_), of which two are specific for powder diffraction (*L*_2_, *L*_3_). The single-crystal Lorentz factor *L*_1_ for the integrated intensity takes into account the intersection between sampling step (the ‘bin’ size in real space) and the finite thickness of the Ewald sphere, and is given as

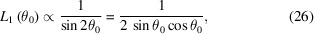

with θ_0_ being the angular position of the Bragg reflection.

In powder diffraction, the second contribution to the Lorentz factor is derived from the angular dependence of the number of observable crystalline particles. The integrated intensity is proportional to the factor

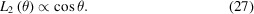

The last contributing factor normalizes the different radii of the Debye–Scherrer rings. The fraction of the diffraction cone that intersects the detector is highest at low angles and at very high angles (backscattering):

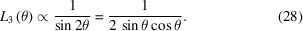

As a result, the Lorentz correction for the area of a powder diffraction peak reads as follows:

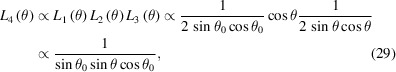

where any constant factor gets absorbed by the overall scale factor. The cosine term in the denominator comes from the conversion between 2θ space and reciprocal (length) space, 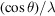 , which is the first term in a Taylor expansion. Such expansion is appropriate to the sampling step, but not to the width of broad peaks. Therefore, the Lorentz factor which should be applied in powder diffraction (Yinghua, 1987 ▸; Warren, 1978 ▸; Ino & Minami, 1984 ▸) is

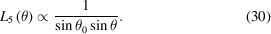

[Ino & Minami (1984 ▸) also show in a rather general way that the powder diffraction intensity of a given Bragg peak should be written with two terms: the main one is the usual line profile (Fourier transform of the CVF), centered on the Bragg position 2θ, and the second term is an identical line profile centered on −2θ. This second term has the effect of avoiding the divergence at 2θ = 0 of the Lorentz 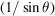 factor.]

For relatively narrow peaks at medium–large 2θ values, this can be simplified to

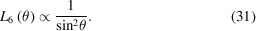

**Figure 10:**
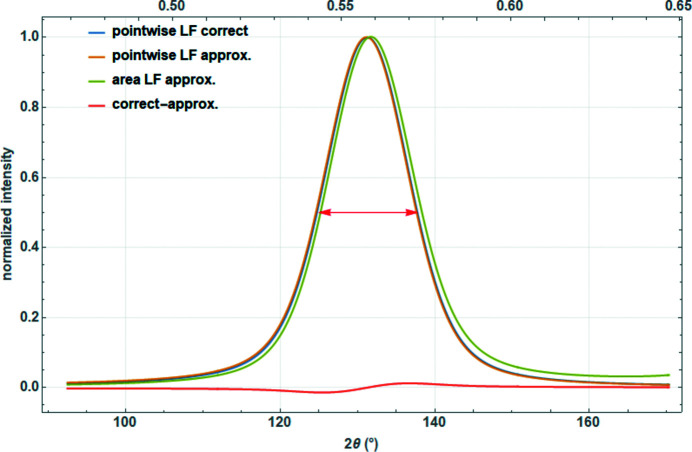
Normalized Voigtian nanocrystalline diffraction peak (FWHM of approximately 13° 2θ) multiplied pointwise by the correct Lorentz factor [equation (30)], by the approximated Lorentz factor [equation (31)] and by the Lorentz factor used for the peak area [equation (29)]. The difference between correct and approximated Lorentz factors is discussed in the text.

The difference between the two definitions [equations (29) and (30)] becomes apparent for nanocrystalline materials where the Bragg peaks span a large range of the diffraction angle, particularly for peaks at high diffraction angles, where the cosine function varies significantly (Fig. 10 ▸). In such cases, peak width, peak shape and peak position change. All in all, equations (29) and (31) are approximations that one should not use in the case of small crystalline domains.

**Figure 11:**
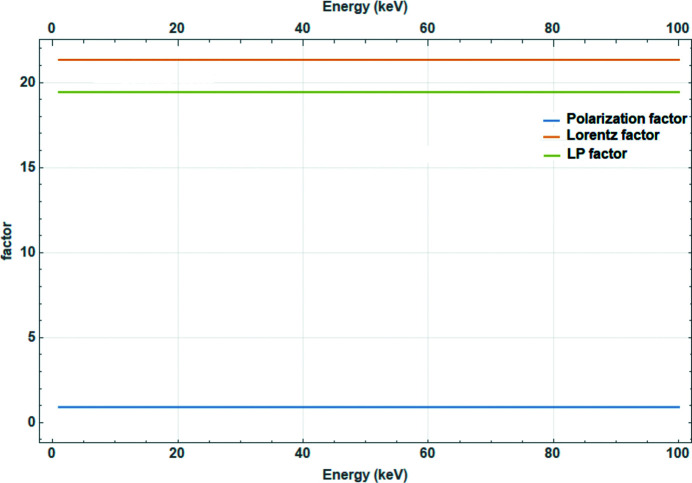
Lorentz and polarization factors for energy-dispersive diffraction.

For energy-dispersive powder diffraction, the Lorentz factor is proportional to λ^3^ (Fig. 11 ▸) and therefore constant. [Note that for neutron time-of-flight (TOF) data the Lorentz factor is proportional to the fourth power of *d* (Zhang *et al.*, 2023 ▸).]

#### 7.2.2. Polarization factor

The purely geometric polarization factor originates from partial polarization of the scattered electromagnetic wave. It is given by the intensity ratio between the diffracted and the primary beam as

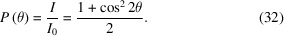

This equation is valid for unpolarized radiation from a laboratory X-ray tube. When a primary or secondary beam monochromator is present, a more general equation is given by

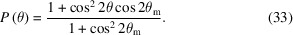

(Not all terms are shown, since the constants are absorbed in a general scale factor when theoretical expressions are used to match experimental data. Which factors are retained depends on which textbook is used. This holds for all expressions in this section.) Here 2θ_m_ is the Bragg angle of the reflection from the monochromator, which is calculated by means of the Bragg equation as

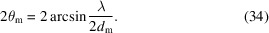

**Figure 12:**
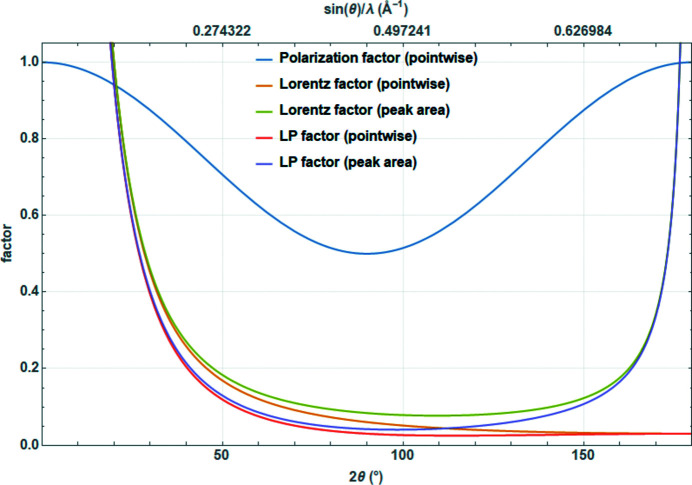
Polarization, Lorentz and combined Lorentz–polarization factors for angular-dispersive laboratory powder diffraction.

The *d* value of the Bragg reflection comes from the monochromator crystal *d*_m_. For unpolarized radiation, 2θ_m_ can be set to 0° (*e.g.* X-ray diffractometers without any monochromator), for fully polarized radiation 2θ_m_ can be set to 90° (*e.g.* synchrotron radiation or constant-wavelength neutron diffraction) (Fig. 12 ▸). In reality, synchrotron radiation is 95–97% polarized. In order to account for fractional polarization of the beam, a factor *K* can be introduced, with *K* = 0.5 for circularly polarized X-rays (*i.e.* laboratory X-ray tubes), *K* = 0 for fully polarized X-rays (ideal synchrotron source) and *K* ∼ 0.05 for a ‘real’ synchrotron source:

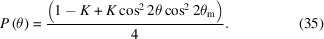

For practical reasons, the Lorentz and polarization factors are often combined into a single Lorentz–polarization factor (Lp factor). For the simplest case of angular-dispersive laboratory X-ray diffraction used for pointwise correction of the powder patterns, using equations (31) and (32) and dropping the ‘2’ in the denominator results in

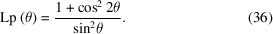

**Figure 13:**
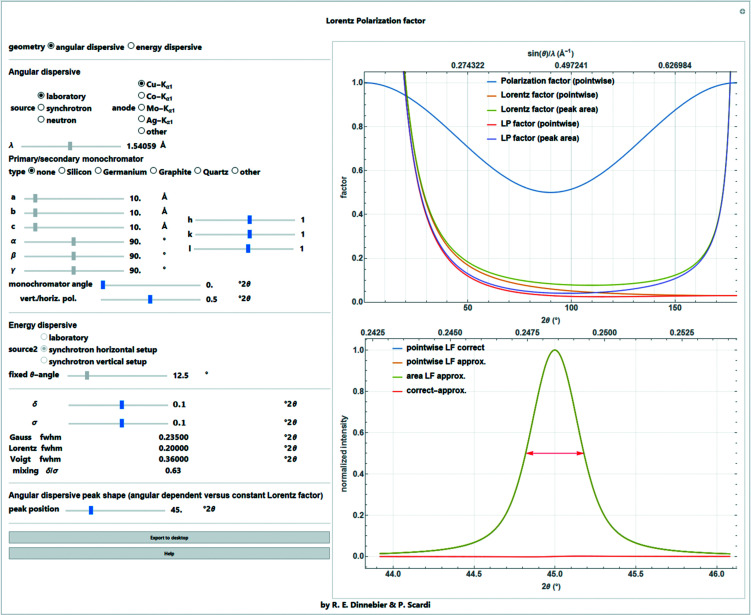
Screenshot of the corresponding *Mathematica* script dealing with Lorentz and polarization factors of different diffraction geometries and sources.

A screenshot of the full *Mathematica* script dealing with all aspects of the Lorentz and polarization corrections for different diffraction geometries is shown in Fig. 13 ▸.

### 7.3. Absorption correction

When passing through matter, X-rays in the keV range are absorbed by both the photoelectric and Compton effects. For accurate powder diffraction work, it is important to consider the effects of absorption on experimental intensities (Maslen, 2006 ▸). Absorption depends on the investigated material, but also on experimental geometry and sample preparation (*e.g.* packing density). Instead of the linear absorption coefficient μ, an effective linear absorption coefficient μ_eff_ should be used to account for the lower packing density of a loose powder. In the following, the correction factors commonly employed in Rietveld analysis for asymmetric/symmetric Bragg–Brentano geometry, Debye–Scherrer, asymmetric/symmetric thin-plate transmission geometry and surface roughness are given.

For direct transmission through a polycrystalline bulk material, the transmitted intensity *I* with respect to the initial intensity *I*_0_ depends on the thickness *t* of the material and its effective linear absorption coefficient  μ_eff_ (Lambert–Beer law):

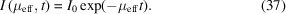

**Figure 14:**
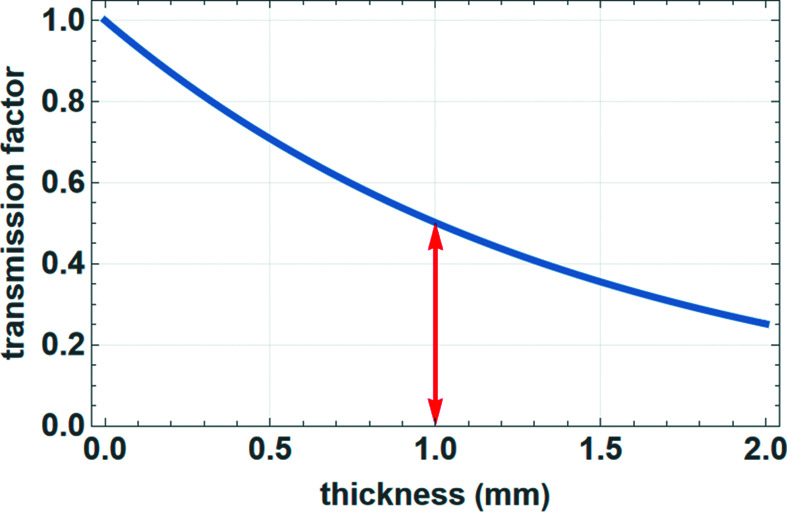
Transmission factor for solid samples in transmission geometry with an absorption coefficient of 6.9 cm^−1^. The red arrow shows the value for a thickness of 1.0 mm (*e.g.* a capillary diameter).

The appropriate absorption correction factor (transmission factor) for calculated intensities (Fig. 14 ▸) therefore is

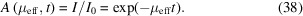

The linear absorption coefficient depends strongly on the energy (wavelength) of the radiation used and also changes rapidly close to the absorption edges.

A comprehensive review of absorption corrections for various diffraction geometries, from which the following formulas were taken, has been given by Rowles & Buckley (2017 ▸). The incoming beam is always approximated as parallel. To calculate the reduction of the diffracted intensity, one must take into account the total path *l* of the incident and the diffracted beams in the sample, and integration must be performed over the entire scattering volume *V* of the sample.

#### 7.3.1. Thin flat-plate transmission geometry

**Figure 15:**
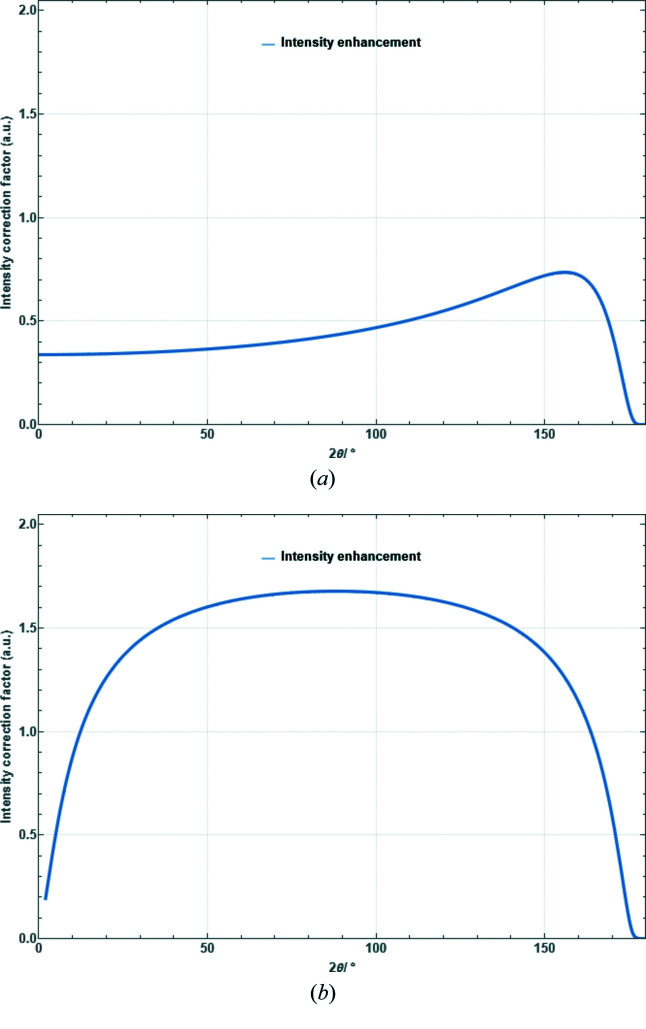
Absorption correction factor for a sample in thin flat-plate transmission geometry as a function of scattering angle for μ_eff_ = 21 mm^−1^ and *t* = 99 µm. (*a*) Symmetric case [equation (41)]. (*b*) Asymmetric case with an angle between the incident beam and specimen surface of 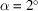 [equation (39)].

For a specimen in asymmetric transmission of thickness *t* with angle α between the incident beam and specimen surface, the correction factor is calculated as [Fig. 15 ▸(*b*)]

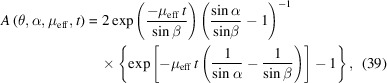

with the angle β between the diffracted beam and the specimen surface defined as

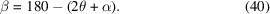

This formula is considerably simpler for symmetric transmission where α = β = 90 − θ [Fig. 15 ▸(*a*)]:

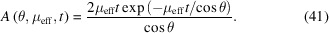




#### 7.3.2. Debye–Scherrer geometry

For cylindrical samples (Debye–Scherrer geometry), the beam must pass through the entire capillary diameter (= two times the radius *R* of the capillary cylinder) at low angles. A reasonable approximation for an absorption correction factor has been given by Sabine *et al.* (1998 ▸):

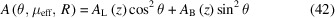

with

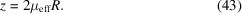

The absorption factors *A*_L_ at the Laue condition ( 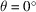 ) and *A*_B_ at the Bragg condition ( 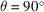 ) are given by

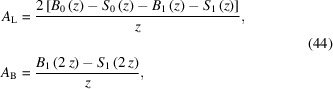

and *B*_*n*_(*z*) is the the modified Bessel function of the first kind, which for integer *n* is defined as

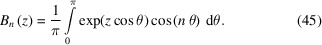

The modified Struve function *S*_*n*_(*z*) is defined as

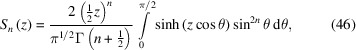

with the gamma function

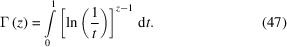

**Figure 16:**
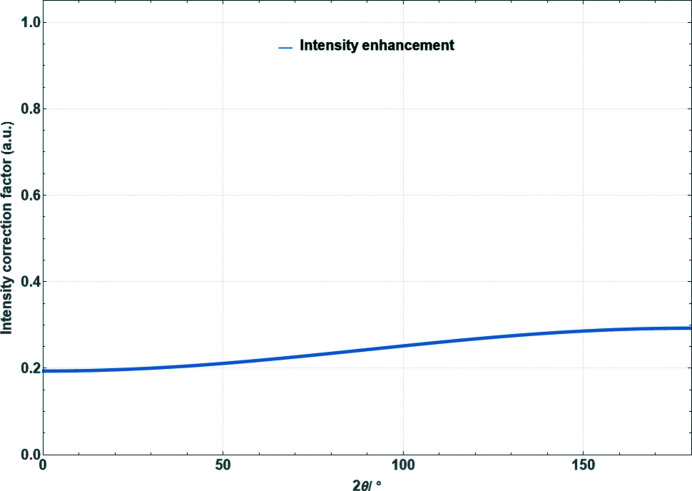
Absorption correction factor for cylindrical samples as a function of scattering angle for μ_eff_ *R* = 1.0 [equation (42)].

Equation (42) gives satisfactory results for μ_eff_ *R* < 10. A typical absorption correction factor for μ_eff_ *R* = 1.0 is shown in Fig. 16 ▸.

A more rigorous treatment using radial symmetry for the calculation of cylindrical absorption coefficients, taking the capillary loading into account, was published by Khalifah (2015 ▸).

#### 7.3.3. Reflection (Bragg–Brentano) geometry

One important consideration in reflection geometry is the requirement that the sample is ‘infinitely thick’, meaning that a negligible fraction of the beam passes straight through the sample. One way to estimate the minimum sample thickness, *t*_min_, required to meet this criterion is to calculate the sample depth required for the incident beam to be reduced to 10^−3^ of its initial intensity. We set 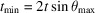 and *I*(surface)/*I*(*t*_min_) = 1000 in equation (37), which simplifies to give

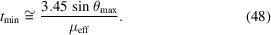

For Ni powder *t*_min_ ≃ 0.013 cm and for a typical organic sample *t*_min_ ≃ 1.3 cm at θ = 90° using Cu *K*α radiation, which makes it unlikely that an organic material will meet the infinite thickness criterion.

**Figure 17:**
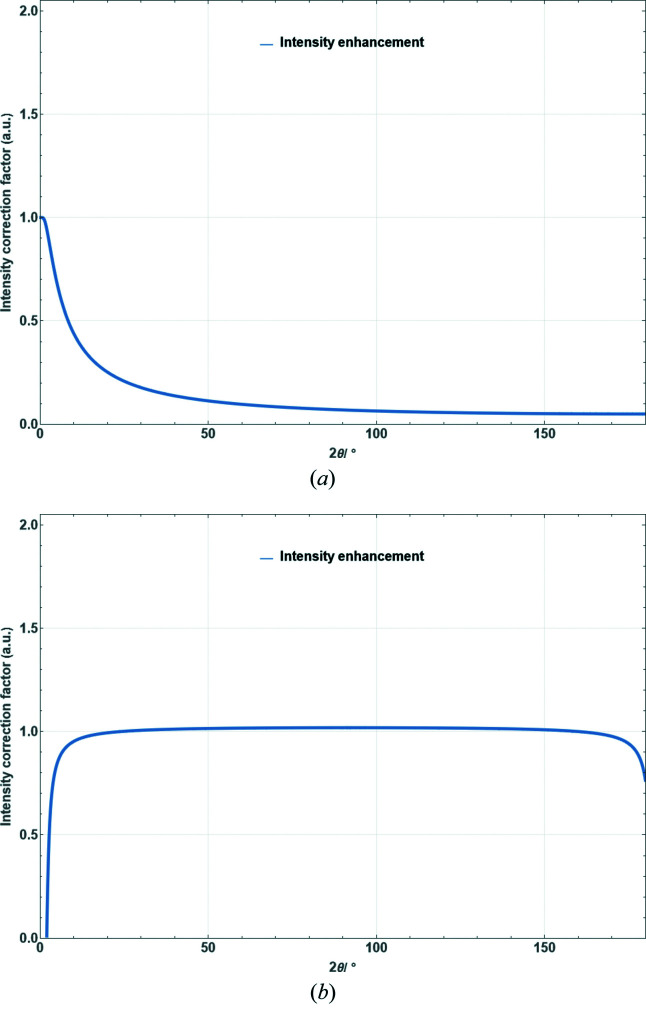
Absorption correction factor for Bragg–Brentano reflection geometry as a function of scattering angle for μ_eff_ = 10 cm^−1^ and *t* = 25 µm. (*a*) Symmetric case [equation (50)]. (*b*) Asymmetric case with an angle between the incident beam and specimen surface of 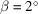 [equation (49)].

For a specimen in asymmetric reflection with angle α between the incident beam and specimen surface, the correction factor is calculated as [Fig. 17 ▸(*b*)]

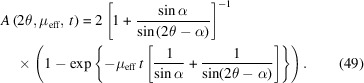

For symmetric reflection α = β = θ (Bragg–Brentano geometry), this simplifies significantly for non-infinitely thick samples to [Fig. 17 ▸(*a*)]

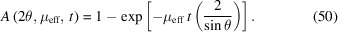




#### 7.3.4. Surface roughness

**Figure 18:**
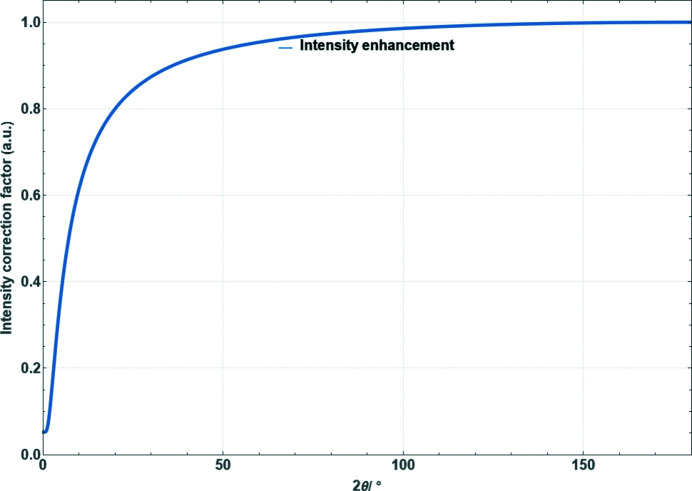
Correction factor for the porosity effect in Bragg–Brentano geometry according to Suortti (1972 ▸) as a function of diffraction angle [equation (51) with *a*_1_ = *a*_2_ = 0.05].

If the packing density in Bragg–Brentano geometry varies with depth, thus creating a ‘rough surface’, the so-called porosity effect reduces the intensity at low Bragg angles. This is also a kind of absorption effect. A common correction function is given by Suortti (1972 ▸):

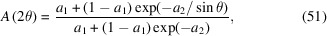

where *a*_1_ and *a*_2_ are refinable parameters (Fig. 18 ▸).

**Figure 19:**
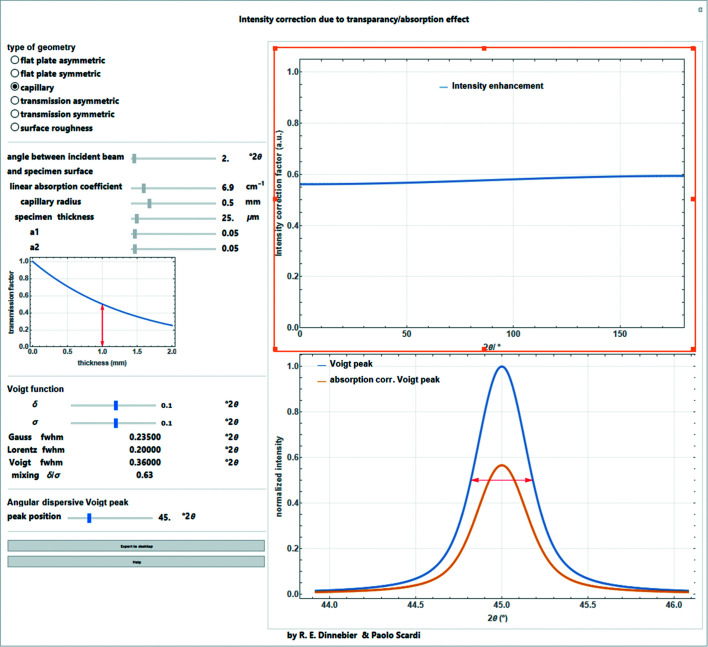
Screenshot of a general *Mathematica* script dealing with different kinds of absorption effects for different diffraction geometries. The intensity modification depending on diffraction angle and the effect on the intensity of a Voigt peak are shown.

A screenshot of the full *Mathematica* script dealing with all aspects of absorption correction for different diffraction geometries and its influence on the intensity of a Bragg peak is shown in Fig. 19 ▸.

### 7.4. Overspill effect

**Figure 20:**
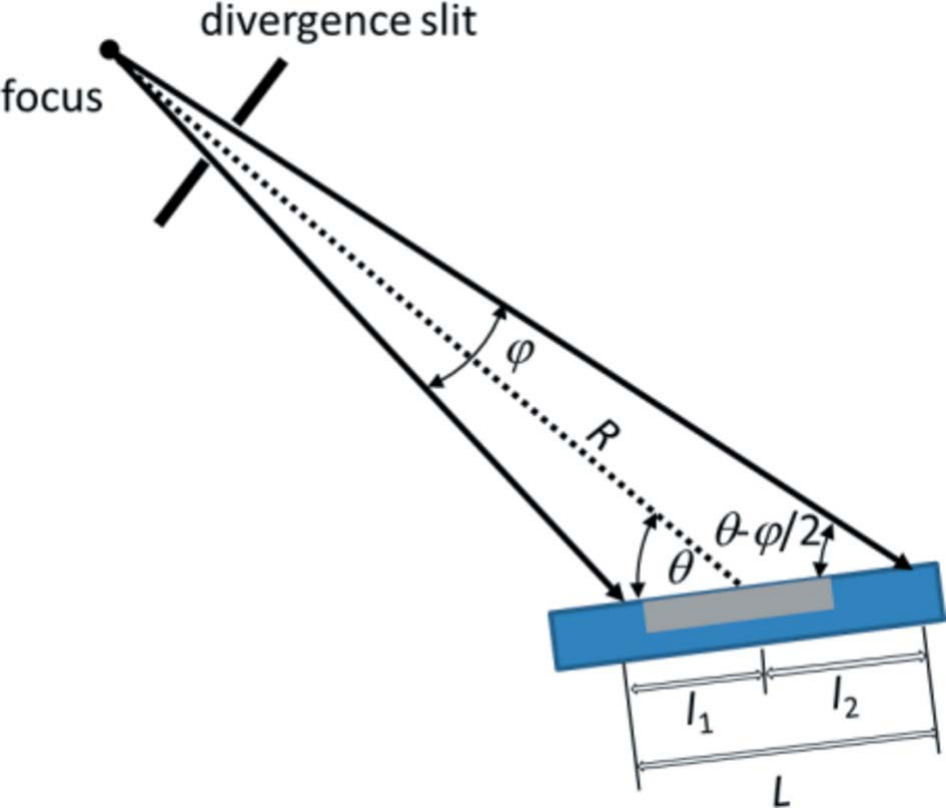
Irradiated length on the surface of a flat-plate sample in Bragg–Brentano geometry for a divergent beam [from Dinnebier *et al.* (2018 ▸ )].

In many diffraction geometries, it is important that the incident beam remains smaller than the sample area at all angles in order to ensure the constant illumination volume condition (in the case of an infinitely thick specimen). This is particularly important in Bragg–Brentano geometry. Nevertheless, at low angles it is common for the irradiated area to become greater than the area covered by the sample on the sample holder. This ‘overspilling’ reduces the intensities up to the diffraction angle at which the two areas are identical (Fig. 20 ▸).

For divergent-beam Bragg–Brentano geometries with a tube opening angle φ, which is determined by the divergence slit, the irradiated length is calculated as (Fischer, 1996 ▸; Krüger & Fischer, 2004 ▸; Pecharsky & Zavalij, 2008 ▸)

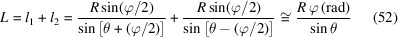

with the goniometer radius *R* (Fig. 20 ▸). The approximation is only valid for very small 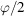 and 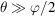 . In the case of small divergence, the beam can be regarded as quasi-parallel and the term *R*φ (rad) corresponds to the thickness of the beam (Fig. 20 ▸).

An intensity correction factor as a function of the diffraction angle can thus be calculated using the approximation in equation (52) for a sample length *S*:

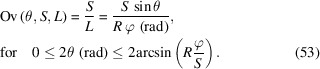

**Figure 21:**
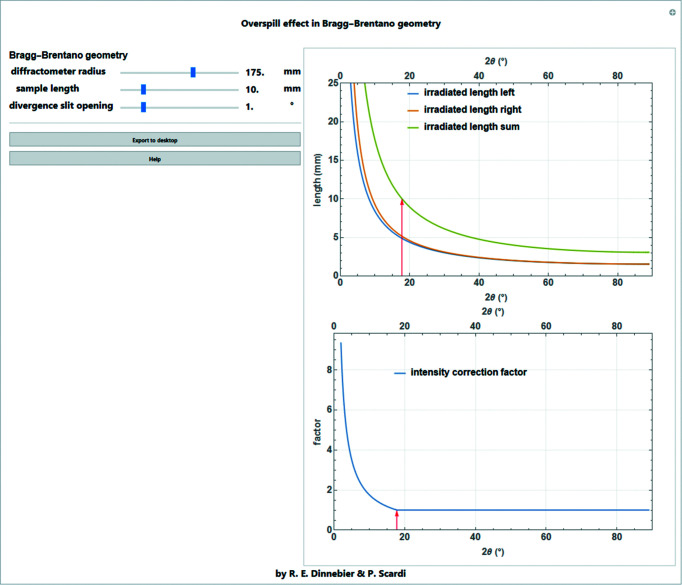
Screenshot of the *Mathematica* script dealing with the overspill effect in Bragg–Brentano geometry, showing the left and right components of the irradiated length and their sum [equation (52)], as well as the corresponding intensity correction factor [equation (53)] for a sample length of 10 mm and an opening of the divergence slit of 1°.

A screenshot of the *Mathematica* script dealing with the overspill effect in Bragg–Brentano geometry, showing the irradiated length and the corresponding intensity correction factor, is shown in Fig. 21 ▸.

## 8. Preferred orientation

Fibers, foils, thin films *etc*. typically show texture due to oriented particles. A similar effect may occur in powders which are not statistically oriented in all directions but show preferred orientation(s). Here we confine our attention to the question of how to account for the expected effects as related to powder diffraction; we do not discuss methods for determining texture. If non-spherical crystallites are prepared in flat-plate sample holders for reflection geometry or between foils for transmission geometry, the crystallites tend to align themselves in one or more preferred orientation(s). If the corresponding lattice planes are in the reflection condition, their intensities are strongly increased. A detailed introduction to this topic is given by Pecharsky & Zavalij (2008 ▸).

The most general way to model the effect of preferred orientation in three dimensions as a complex radial distribution is a symmetry-adapted spherical harmonic expansion (Bunge, 1982 ▸). The spherical harmonic functions can be expanded in a series to describe, in principle, any direction (θ, φ) dependent function. θ and φ are the coordinates of a spherical surface representing the spherical coordinates of the reciprocal-lattice vector **s** of each Bragg reflection normal to the *hkl* plane. They are similar to latitude and longitude except that θ goes from 0 to π and φ goes from 0 to 2π. In general, the spherical harmonics *Y*_*lm*_(θ, φ) are a complete and orthogonal set of solutions of the angular part of Laplace’s equation in three dimensions. Since the spherical harmonic functions are orthogonal, the integral of the product of two different harmonics over the surface of the sphere is zero. For powder diffraction, the symmetrized and normalized real spherical harmonics of even order (due to the inversion center introduced by diffraction) are the most important. The functions are normalized such that the maximum value of each component is unity (Järvinen, 1993 ▸). Therefore, the simplest normalized spherical harmonic represents a unity sphere:

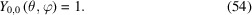

**Figure 22:**
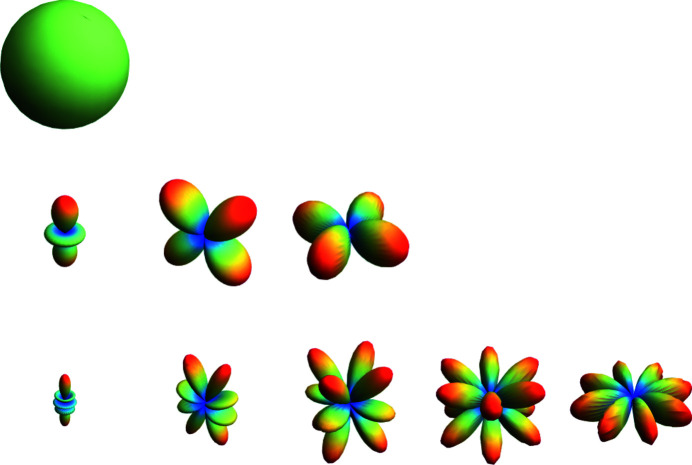
Graphical visualization of the normalized real components of zero- [*Y*_0, 0_(θ, φ) top], second- [*Y*_2, 0_(θ, φ), *Y*_2, 1_(θ, φ), *Y*_2, 2_(θ, φ) middle] and fourth- [*Y*_4, 0_(θ, φ),  *Y*_4, 1_(θ, φ),  *Y*_4, 2_(θ, φ),  *Y*_4, 3_(θ, φ), *Y*_4, 4_(θ, φ) bottom] order spherical harmonics.

The normalized real components of second-order spherical harmonics become

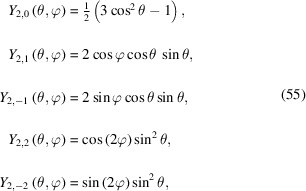

which are visualized in Fig. 22 ▸.

**Figure 23:**
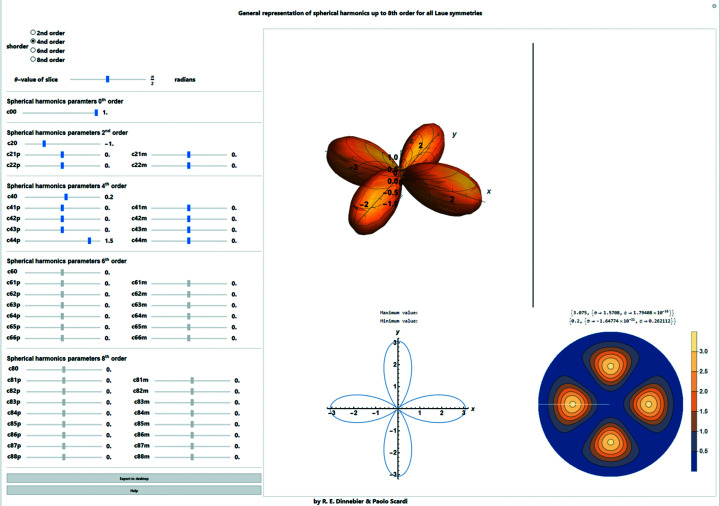
Screenshot of the general representation of symmetry-adapted spherical harmonics up to eighth order for all Laue symmetries as used *e.g.* to describe preferred orientation in reciprocal space. The graphics show the three-dimensional representation, a two-dimensional slice at a given θ value and the two-dimensional spherical projection.

The preferred orientation factor *T_hkl_* is proportional to the probability of the point of the reciprocal lattice, *hkl*, being in the reflecting position (*i.e.* the probability of being located on the surface of the Ewald sphere). In other words, this multiplier is proportional to the number of crystallites with *hkl* planes parallel to the surface of the flat sample:

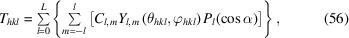

where *C*_0,0_ = 1, *C*_*l*,*m*_ are Rietveld refinable parameters, *Y*_*l*,*m*_(θ_*hkl*_, φ_*hkl*_) are the symmetry-adapted spherical harmonics of even order *L*, α corresponds to the angle in degrees between the polar axis and the scattering vector, and 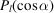 is a Legendre polynomial (Järvinen, 1993 ▸). For the most common case of symmetric reflection, α = 0 and *P*_*l*_(1) = 1. In practice, orders higher than *L* = 8 are rarely used (Fig. 23 ▸).

## 9. Intensity distribution in a powder pattern

The intensity distribution in a powder pattern is strongly influenced by the size and shape of the crystallites. In principle, there are two different approaches for calculation: (i) assuming an ensemble of identical crystallites, with a given CVF involving a structure factor, or (ii) considering the scattering from the total atom-pair distribution in a discrete crystallite (Debye scattering equation). The intensities are then modified by the correction factors described above.

## 10. Common volume function

The intensity from a powder sample made of *M* identical crystallites can be written as (Ino & Minami, 1984 ▸; Beyerlein *et al.*, 2011 ▸)

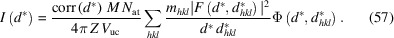

Here, *N*_at_ is the number of atoms in a crystallite and *V*_uc_ the unit-cell volume; *Z* is the number of formula units in the unit cell; *m_hkl_* is the multiplicity (structure factor, multiplicity, Lorentz factor, polarization factor, absorption and temperature effects are explained in detail in this paper) of the Bragg reflection with the Miller indices *hkl*; corr(d*) is a known function of d*, typically including effects of polarization, absorption and temperature; 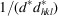 is the Lorentz factor of equation (30), 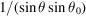 , including the constant term λ^2^/4; 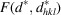 is the structure factor of equation (17), explicitly showing the dependence on d*, for the form factor, and on 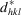 , for the phase term. This double dependence is consistent with the ‘random shift treatment’ of Ino & Minami (1979 ▸); the powder actually consists of crystallites with the same size and shape, each obtained from different shifts of the unit cell relative to the center of mass of the shape [see also Scardi *et al.* (2011 ▸)]. 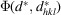 is the line profile function for the size effect [in this work, we restrict the contribution for the line profile to the size effect; for instrumental contributions and the sample-related microstructural effect, see Part 1 (Dinnebier & Scardi, 2021 ▸)] normalized to unit area:

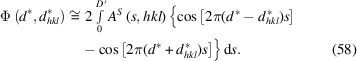

Φ depends on *D*′, the maximum length along the given [*hkl*] direction in the crystallite, and on *A* ^*S*^, the CVF: the volume of the intersection between the crystalline domain and the same domain translated along [*hkl*] by a distance *S*, normalized by the domain volume. As first shown by Patterson (1939 ▸) and then by Warren (1978 ▸), equation (58) is exact for a spherical domain shape, whereas it is just an approximation for any other shape. As shown by Ino & Minami (1984 ▸), equation (58) is the main term of a series expansion, and the terms following this are necessary only for small, non-spherical crystalline domains.

Equations (57) and (58) implicitly assume that the domain volume, *V*_0_, can be written as

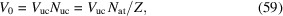

where *N*_uc_ is the number of unit cells in the domain. This is clearly an approximation, considering the inherently discrete nature of nanocrystals; but equation (59) makes equations (57) and (58) formally identical to equations (17) and (19) of Beyerlein *et al.* (2011 ▸), apart from *M*, the number of crystallites in the powder, which appears in equation (57) but not in the work of Beyerlein *et al.* (2011 ▸).

The second cosine in equation (58) gives a minor contribution and is negligible unless the domain sizes are very small (a few nm); it is however correct and useful to eliminate the divergence of intensity at d* = 0, due to the Lorentz factor [ 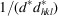 ]. In most practical applications, where crystalline domains are not very small and patterns are recorded for d* >> 0, it is possible to neglect the second cosine and write

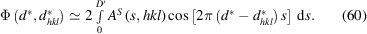

Equation (61), first proposed by Stokes & Wilson (1942 ▸), was obtained within the tangent plane approximation (TPA) originally proposed by Laue (1926 ▸). As shown in Part I, equation (73) (Dinnebier & Scardi, 2021 ▸), for a spherical domain of diameter *D*,

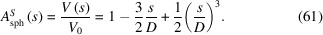

Equation (60) can be integrated using equation (61). Considering that in this case *D*′ = *D*, integration is straightforward and gives

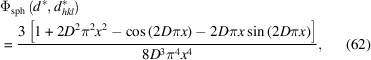

where we set 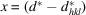 , the distance from the Bragg position in reciprocal space. The complete expression from equation (58) is

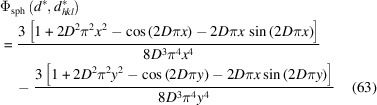

with 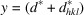 .

**Figure 24:**
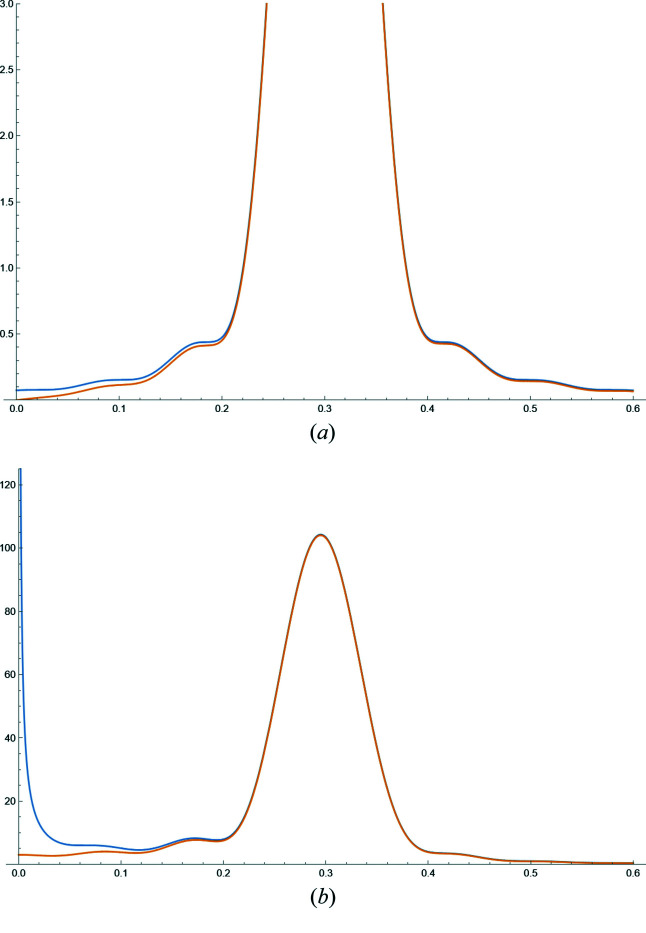
(*a*) Comparison between equations (62) (blue) and (63) (orange) for a powder of spherical domains of diameter *D*_s_ = 12.4 and 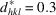 (units are defined according to those of the abscissa d*, *e.g.* nm/nm^−1^ or Å/Å^−1^); (*b*) same comparison but multiplied by the Lorentz factor [ 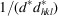 ]. The second term in equation (64) avoids the divergence for d* = 0.

Fig. 24 ▸ shows a comparison between the TPA equations (62) and (63) for a powder of spherical domains. As already pointed out, equation (63) avoids the divergence due to the Lorentz factor of equation (30).

For a domain of cubic shape and edge *D*, the normalized CVF reads

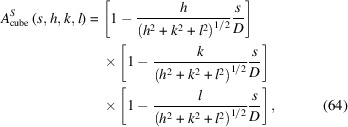

where the Miller indices must be chosen such that *h* ≥ *k* ≥ *l*. The integration limit in equations (58) and (60), in this case, is

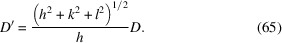

Then, equation (60) gives the following result for the normalized line profile:

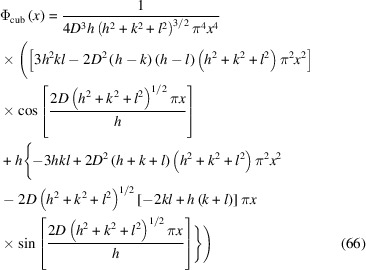

with 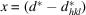 , and always assuming *h ≥ k ≥ l*.

**Figure 25:**
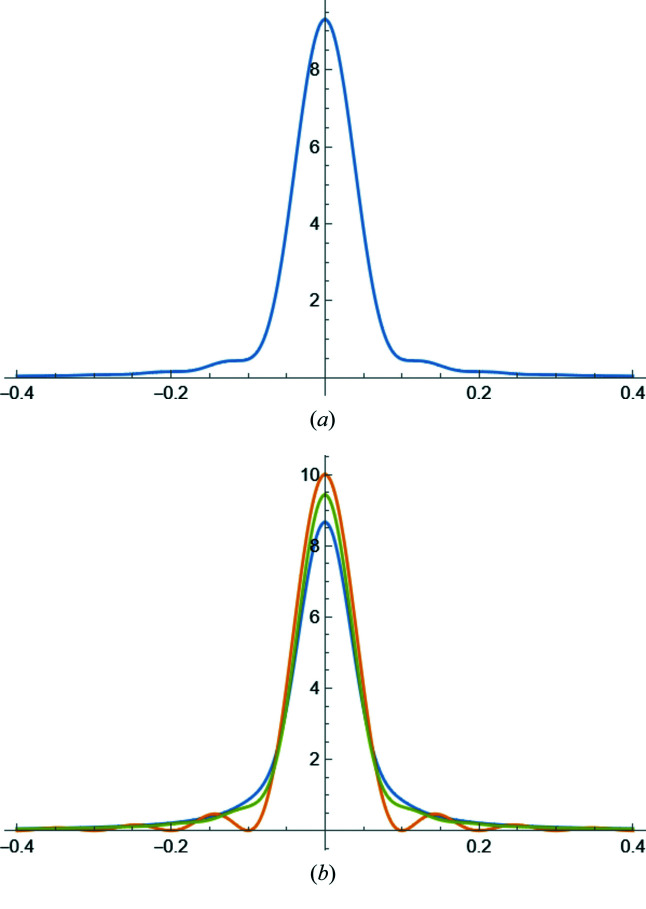
Line profile functions for a powder of (*a*) spherical crystallites and (*b*) cubic crystallites: (111) blue; (200) orange; (220) green. The diameter of the sphere is chosen to have the same volume as the cube: *D*_c_ = 10 and *D*_s_ = 12.4. The abscissa is 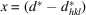 . Units are coherent, *i.e.* if *D*_c_, *D*_s_ are in nm or Å, *x* is in nm^−1^ or Å^−1^.

Examples of line profile functions are shown in Fig. 25 ▸: equation (62) for a powder of spherical crystallites [line profiles are identical for any (*hkl*) in d* space] in (*a*), and equation (66) for a powder of cubic crystallites of edge *D*_c_, for different (*hkl*)s in (*b*). The sphere diameter is 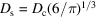 , so that the sphere and cube have the same volume.

## 11. Debye scattering equation

The Debye scattering equation (DSE) gives the powder pattern intensity from a single crystallite consisting of *N*_at_ atoms:

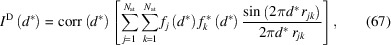

where *f* is the atomic scattering factor and *r_jk_* the distance between any two atoms in the scattering domain. Derivations of the Debye formula can be found in the work of Debye (1915 ▸) or in textbooks, *e.g.* Warren (1990 ▸), Guinier (1963 ▸) and Dinnebier *et al.* (2018 ▸). Note that the Lorentz factor is automatically included in the formalism. For simplicity, if we refer to a single-element phase, like a metal, the DSE can be written as

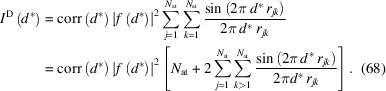

The DSE is computationally demanding due to the double sum that appears in equations (67) and (68), which involves the computation of 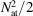 terms for each step of d*. For practical purposes, a viable approximation first proposed by Germer & White (1941 ▸) is binning similar distances. This reduces the complexity to a single sum:

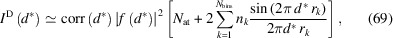

where a histogram of distances for each pair of atomic species is calculated first and then divided into *N*_bins_ bins with *n*_*k*_ the number of pairs of distance *r*_*k*_ corresponding to the *k*th bin. This approach can easily be encoded in programs like *Debyer* (Wojdyr, 2011 ▸).

## 12. Comparison of DSE and CVF for copper nanoparticles

**Figure 26:**
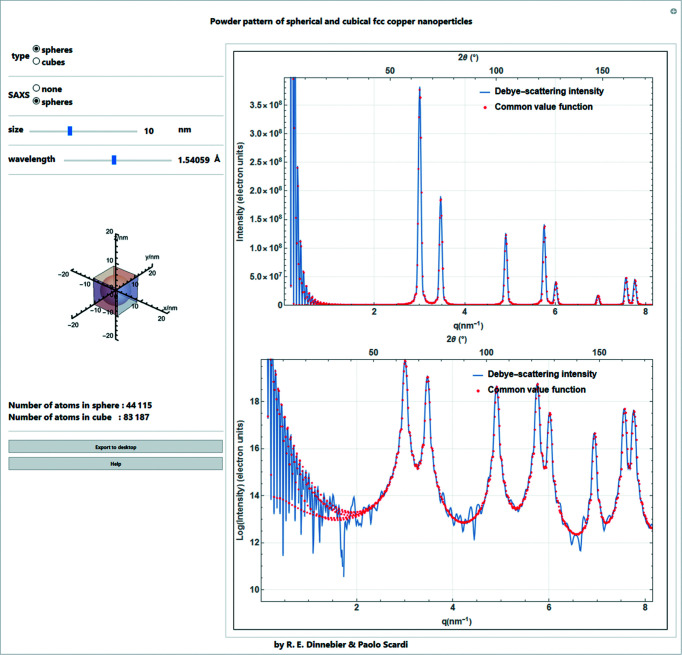
Screenshot of a *Mathematica* script for simulation of powder patterns on linear and log scales using the DSE [calculation done by *Debyer* (Wojdyr, 2011 ▸)] and CVF for f.c.c. Cu nanoparticles (unit-cell parameter *a* = 3.615 Å) of different sizes (from 1 to 25 nm) and shapes (spheres, cubes) with or without the SAXS term for spheres. (The visible fluctuations in the DSE pattern, which are amplified by the logarithmic scale, are the result of limits in the numerical precision used in the simulation software.)

In the following *Mathematica* script, powder patterns are simulated using DSE and CVF for f.c.c. Cu nanoparticles (unit-cell parameter *a* = 3.615 Å) of different sizes (from 1 to 25 nm) and shapes (spheres, cubes) (Fig. 26 ▸). The standard output of the *Debyer* software used to simulate the powder patterns with the DSE is (*q*, *I*/*N*_at_), that is *q* = 2πd* in Å^−1^ and the intensity/number of atoms in the crystallite. Expressions derived from the CVF presented above, equation (57), give the intensity for a powder of *M* crystallites. Therefore, to compare patterns the DSE intensity is multiplied by *N*_at_, while *M* = 1 in equation (57). For both expressions corr(d*) = 1, as we assume linear polarization. In the *Mathematica* script, equation (57) is written for 2θ, with a wavelength λ = 1.5406 Å, or for *q* in Å^−1^. The structure-factor square modulus in equation (57) can be written as 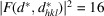 |*f*(d*)|^2^, and *Z* = 4 for the f.c.c. unit cell. Then equation (57) is

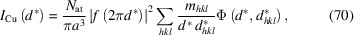

with 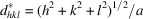 , or

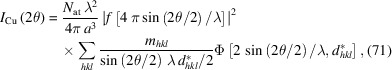

where 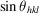 is written as 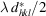 , or

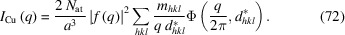

The DSE and CVF can never give identical results. The DSE is intrinsically based on a discrete structure, and is used for a well defined nanoparticle, in which the coordinates of all the atoms are known. On the other hand, the CVF approach, with the ‘random shift treatment’ of Ino & Minami (1984 ▸), generates a pattern referring to an average of nominally similar but not identical nanoparticles. Additionally, the DSE generates the small-angle X-ray scattering (SAXS) signal for the discrete nanoparticle. However, for practical purposes, besides typically having a distribution of different shapes/sizes, the nanoparticles are packed together in powdered samples, thus modifying or destroying the SAXS components that might otherwise be observed for monodisperse crystallites in dilute solution.

Nevertheless, in order to compare the DSE and CVF approaches for a powder of identical nanocrystals, a SAXS contribution can be added to equation (57). For a sphere, the Rayleigh formula (Rayleigh, 1910 ▸) is

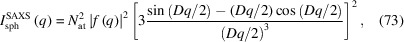

whereas for a cubic shape

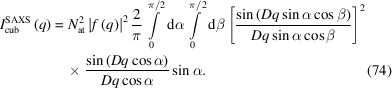

Equation (74) is computationally demanding, and increasingly so for larger nanoparticles.

Equations (57), (63) and (73) should provide a near-perfect match with the DSE result for spherical domains. The agreement is much lower for cubic nanoparticles for which equation (58) provides only a first-order approximation. However, it is important to underline that what is shown in this last part concerns powders and polycrystalline materials with crystalline domains at the nanoscale, and is really useful and necessary when these domains have a well defined shape. In all other cases, and even when microstructural information is not of primary interest, the empirical approach based on adaptive functions, such as Voigt curves, represents a valid and much simpler alternative.
